# Supplementary material for: An inherited mitochondrial DNA mutation remodels inflammatory cytokine responses in macrophages and in vivo in mice
Source: Nat Commun. 2025 Nov 20;16:10222. doi: 10.1038/s41467-025-65023-4 (PMC12635290; doi:10.1038/s41467-025-65023-4)
Supplement: Supplementary file 1 — Supplementary Information [file 41467_2025_65023_MOESM1_ESM.pdf]

Inventory of Supplementary Information (SI)

An inherited mitochondrial DNA mutation remodels inflammatory cytokine responses in macrophages and *in vivo*

Eloïse Marques[1], Stephen P. Burr[1], Alva M. Casey[1], Richard J. Stopforth[2,3], Chak Shun Yu[1], Keira Turner[1], Dane M. Wolf[1], Marisa Dilucca[4], Vincent Paupe[1], Suvagata Roy Chowdhury[1], Victoria J. Tyrrell[5], Robbin Kramer[1], Yamini M. Kanse[1], Chinmayi Pednekar[6], Chris A. Powell[1], James B. Stewart[7], Julien Prudent[1], Michael P. Murphy[1], Michal Minczuk[1,8], Valerie B. O'Donnell[5], Clare E. Bryant[4], Patrick F. Chinnery[1,8], Arthur Kaser[2,3], Alexander von Kriegsheim[6], Dylan G. Ryan[1,9#]

[1]MRC Mitochondrial Biology Unit, School of Clinical Medicine, University of Cambridge, Cambridge Biomedical Campus, Cambridge, UK.
[2]Cambridge Institute of Therapeutic Immunology and Infectious Disease, Jeffrey Cheah Biomedical Centre, University of Cambridge, Cambridge CB2 0AW, UK.
[3]Division of Gastroenterology and Hepatology, Department of Medicine, University of Cambridge, Addenbrooke's Hospital, Cambridge CB2 0QQ, UK.
[4]Department of Medicine, Addenbrooke's hospital, Cambridge Biomedical Campus, Cambridge, UK.
[5]Division of Infection and Immunity, School of Medicine, Cardiff University, Cardiff, UK.
[6]Cancer Research UK Centre, Institute of Genetics and Cancer, University of Edinburgh, UK.
[7]Biosciences Institute, Faculty of Medical Sciences, Newcastle University, Newcastle upon Tyne, UK.
[8]Department of Clinical Neurosciences, School of Clinical Medicine, University of Cambridge, Cambridge Biomedical Campus, Cambridge, UK.
[9]School of Biochemistry and Immunology, Trinity Biomedical Sciences Institute, Trinity College, Dublin 2, Ireland.

[#]Corresponding author

E-mail address: RYAND67@tcd.ie

Supplementary figures:

- Supplementary figure 1 – Reduced mitochondrial respiration in *m.5019A>G* macrophages
- Supplementary figure 2 – Increased mitoribosome subunits and decreased OxPhos in *m.5019A>G* macrophages
- Supplementary figure 3 – Reduced pyruvate entry into the TCA cycle in *m.5019A>G* macrophages
- Supplementary figure 4 – TCA cycle remodelling and U-^{13}C-glutamine and-glucose tracing in *m.5019A>G* macrophages
- Supplementary figure 5 – U-^{13}C-glutamine and -glucose tracing into the AAS in *m.5019A>G* macrophages
- Supplementary figure 6 – Increased type I IFN signalling and reduced IL-1β and COX2 levels in *m.5019A>G* macrophages
- Supplementary figure 7 – Reduced *Il6* expression and IL-6 release in *m.5019A>G* macrophages
- Supplementary figure 8 – Mitochondrial network remodelling in *m.5019A>G* macrophages
- Supplementary figure 9 – Early phase type I IFN in *m.5019A>G* macrophages is independent of mitochondrial nucleic acids
- Supplementary figure 10 – Elevated type I IFN levels in *m.5019A>G* mice

Supplementary tables:

- Supplementary Table 1 – Pyrosequencing primers
- Supplementary Table 2 – ddPCR primers
- Supplementary Table 3 – Antibodies used for all methods
- Supplementary Table 4 – RT-qPCR primers

Supplementary figure 1

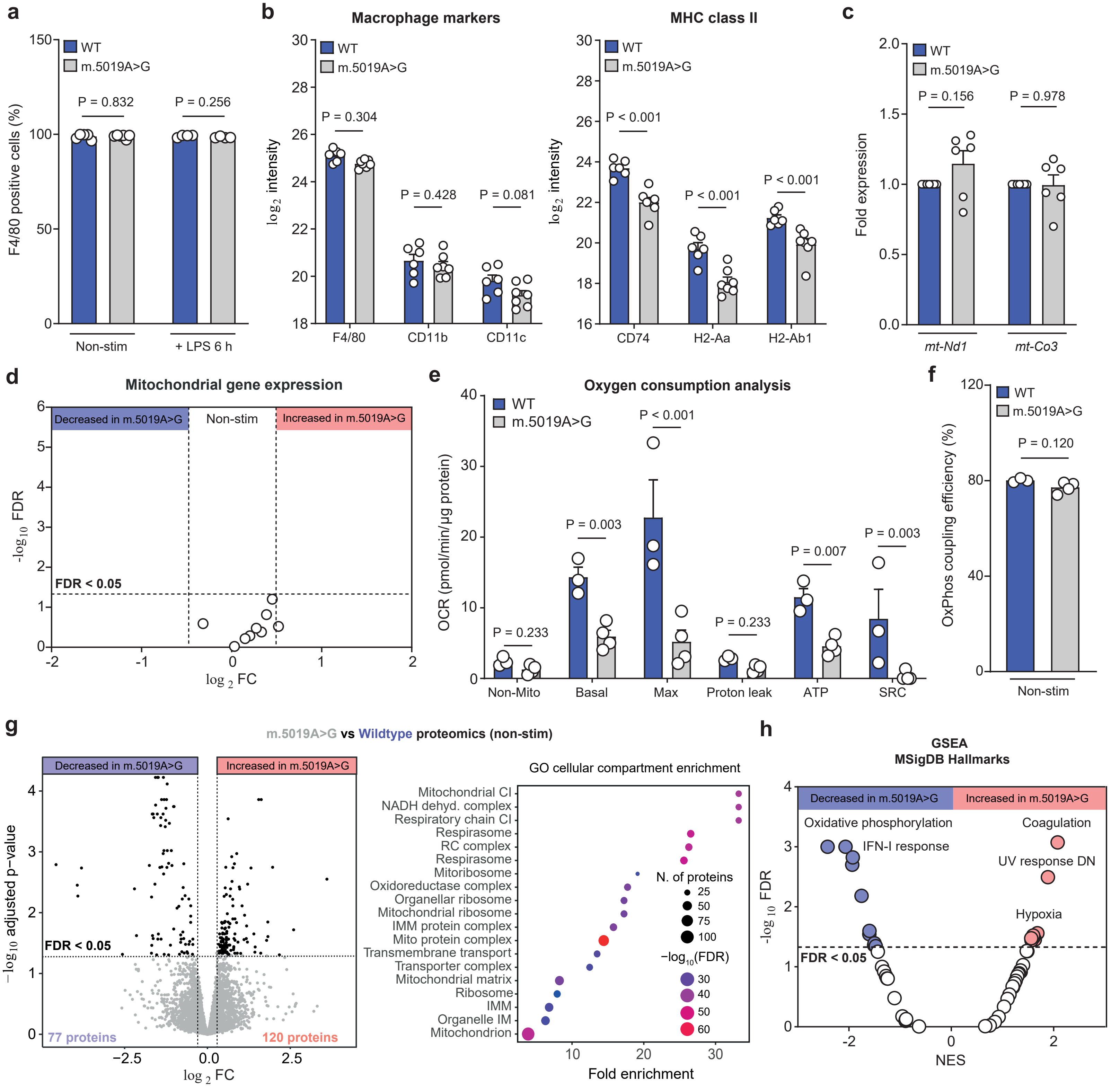

Supplementary figure 1 – Reduced mitochondrial respiration in *m.5019A>G* macrophages

a, F4/80 cell surface staining of non-stimulated (non-stim) and lipopolysaccharide (LPS)-stimulated wildtype (WT) and *m.5019A>G* BMDMs (n = 5; LPS 6 h). **b,** Proteomic analysis of F4/80, CD11b and CD11c (left), and H2-Aa (P = 0.000034), H2-Ab1 (P = 0.000471) and CD74 (P = 0.000034) (right), in non-stim WT and *m.5019A>G* (n = 6-7) BMDMs. **c**, *mt-Nd1* and *mt-Co3* expression in non-stim WT and *m.5019A>G* BMDMs (n = 6). **d,** Volcano plot of mitochondrial genes from RNA sequencing (n = 3). **e-f**, Seahorse XFe24 oxygen consumption rate (OCR) analysis in non-stim WT (n = 3) and *m.5019A>G* (n = 4) BMDMs (P = 0.00000039). **g,** Volcano plot (left) and Overrepresentation analysis (ORA) using gene ontology (GO) cellular compartment terms (right) of all differentially abundant proteins identified in non-stim *m.5019A>G* vs WT BMDMs (n = 6; WT and n = 7; *m.5019A>G*). **h,** Gene set enrichment analysis (GSEA) of RNA sequencing from non-stim WT and *m.5019A>G* BMDMs (n = 3). Data are mean ± s.e.m. n number represents independent biological replicates (mice) from a minimum of two independent experiments. P values calculated using two-tailed Student’s t-test for two group comparisons or multiple two-tailed unpaired t-tests corrected for multiple comparisons using Benjamini, Krieger and Yekutieli method.

Supplementary figure 2

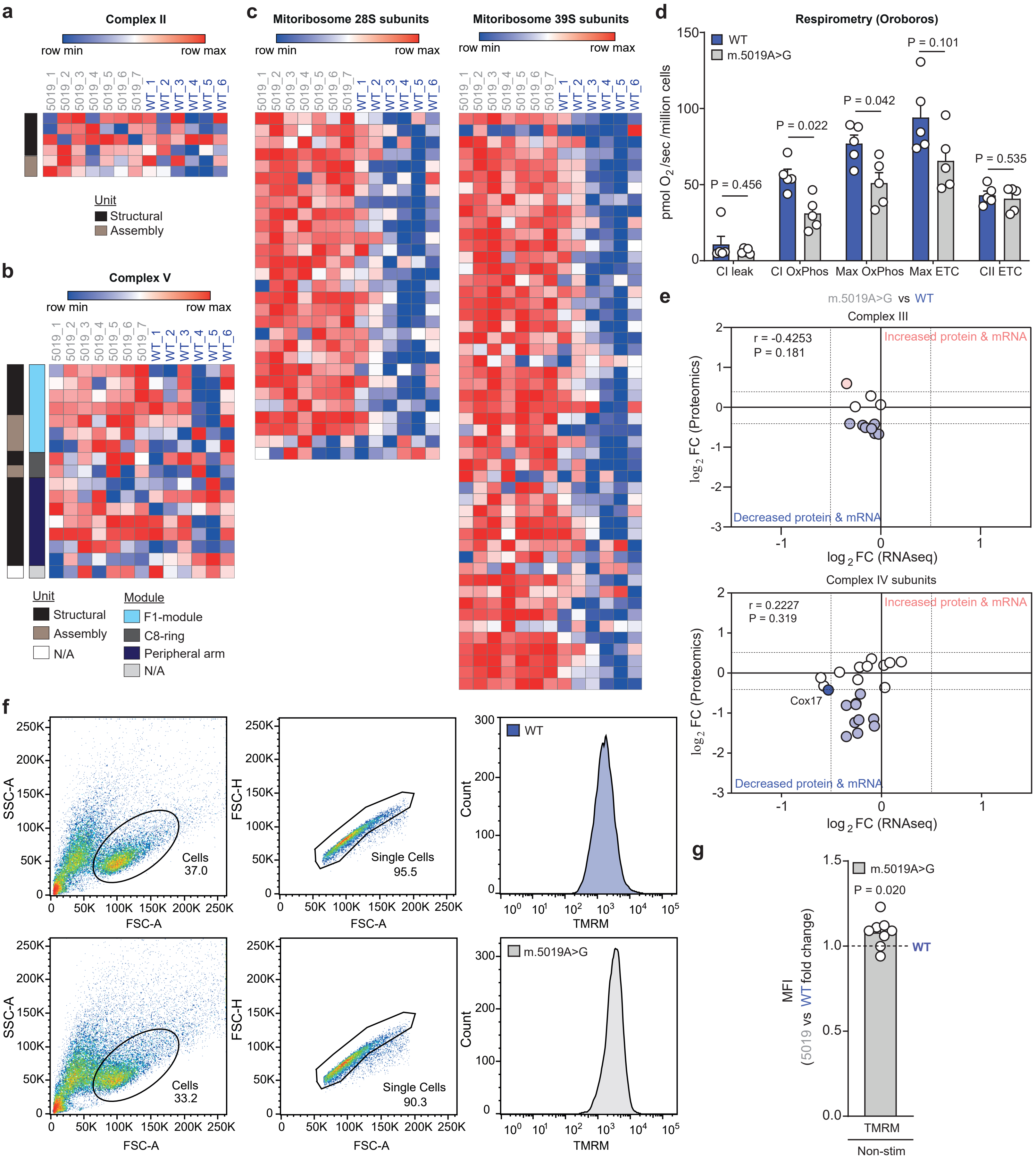

Supplementary figure 2 – Increased mitoribosome subunits and decreased OxPhos in *m.5019A>G* macrophages

a-c, Heatmap of complex II (CII), ATP synthase (CV), and mitoribosome 28S and 39S subunits in non-stimulated (non-stim) wildtype (WT) and *m.5019A>G* BMDMs (*n* = 6-7). **d,** Oroboros respirometry analysis of permeabilised non-stim WT and *m.5019A>G* BMDMs (*n* = 5). ETC = electron transfer capacity. **e,** Comparison of $\log_2$FC values of CIII and CIV subunits from proteomics (*n* = 6; WT and *n* = 7; *m.5019A>G*) and RNA sequencing (*n* = 3) data with Pearson *r* correlation and statistical analysis applied. **f-g,** Flow cytometry gating strategy (representative schematic) and unnormalised tetramethyl rhodamine methyl ester (TMRM) signal in non-stim *m.5019A>G* vs WT BMDMs (*n* = 8). Data are scaled $\log_2$ intensities, $\log_2$FC or mean ± s.e.m. *n* number represents independent biological replicates (mice) from a minimum of three independent experiments. *P* values calculated using two-tailed Student's t-test for two group comparisons or multiple two-tailed unpaired t-tests corrected for multiple comparisons using Benjamini, Krieger and Yekutieli method.

Supplementary figure 3

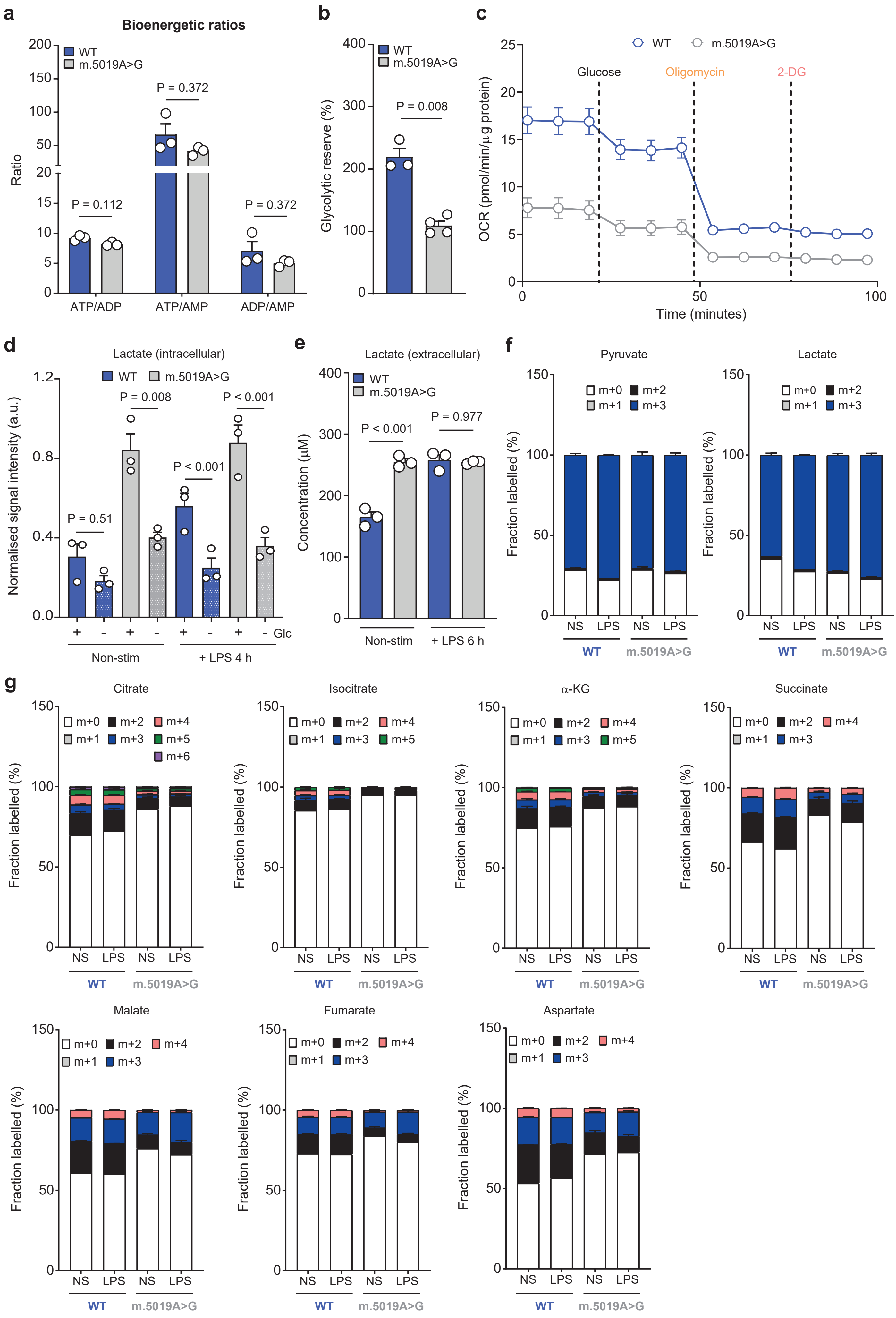

Supplementary figure 3 – Reduced pyruvate entry into the TCA cycle in *m.5019A>G* macrophages

a, ATP/ADP, ATP/AMP and ADP/AMP ratios in non-stimulated (non-stim) wildtype (WT) and *m.5019A>G* BMDMs (*n* = 3). **b-c,** Glycolytic reserve and oxygen consumption rate (OCR) analysis from Glycostress test in non-stim WT (*n* = 3) and *m.5019A>G* (*n* = 4) BMDMs. **d,** Intracellular lactate measurements from metabolomics in non-stim and LPS-stimulated WT and *m.5019A>G* BMDMs in the presence or absence of glucose (Glc) (*n* = 3; LPS 4 h) (*P* = 0.000319; *P* = 0.000052). **e,** Extracellular lactate measurements using Lactate-Glo^{TM} assay in non-stim and LPS-stimulated WT and *m.5019A>G* BMDMs (*n* = 3; LPS 6 h) (*P* = 0.0000594). **f-g**, Total isotopologue distribution (% fraction labelling) of pyruvate, lactate, TCA cycle metabolites and aspartate from U-^{13}C-glucose tracing in non-stim and LPS-stimulated WT and *m.5019A>G* BMDMs (*n* = 5; LPS 6 h). Data are mean ± s.e.m. *n* number represents independent biological replicates (mice) from a minimum of two independent experiments. *P* values calculated using two-tailed Student's t-test for two group comparisons or multiple two-tailed unpaired t-tests corrected for multiple comparisons using Benjamini, Krieger and Yekutieli method or one-way ANOVA corrected for multiple comparisons using Tukey method.

Supplementary figure 4

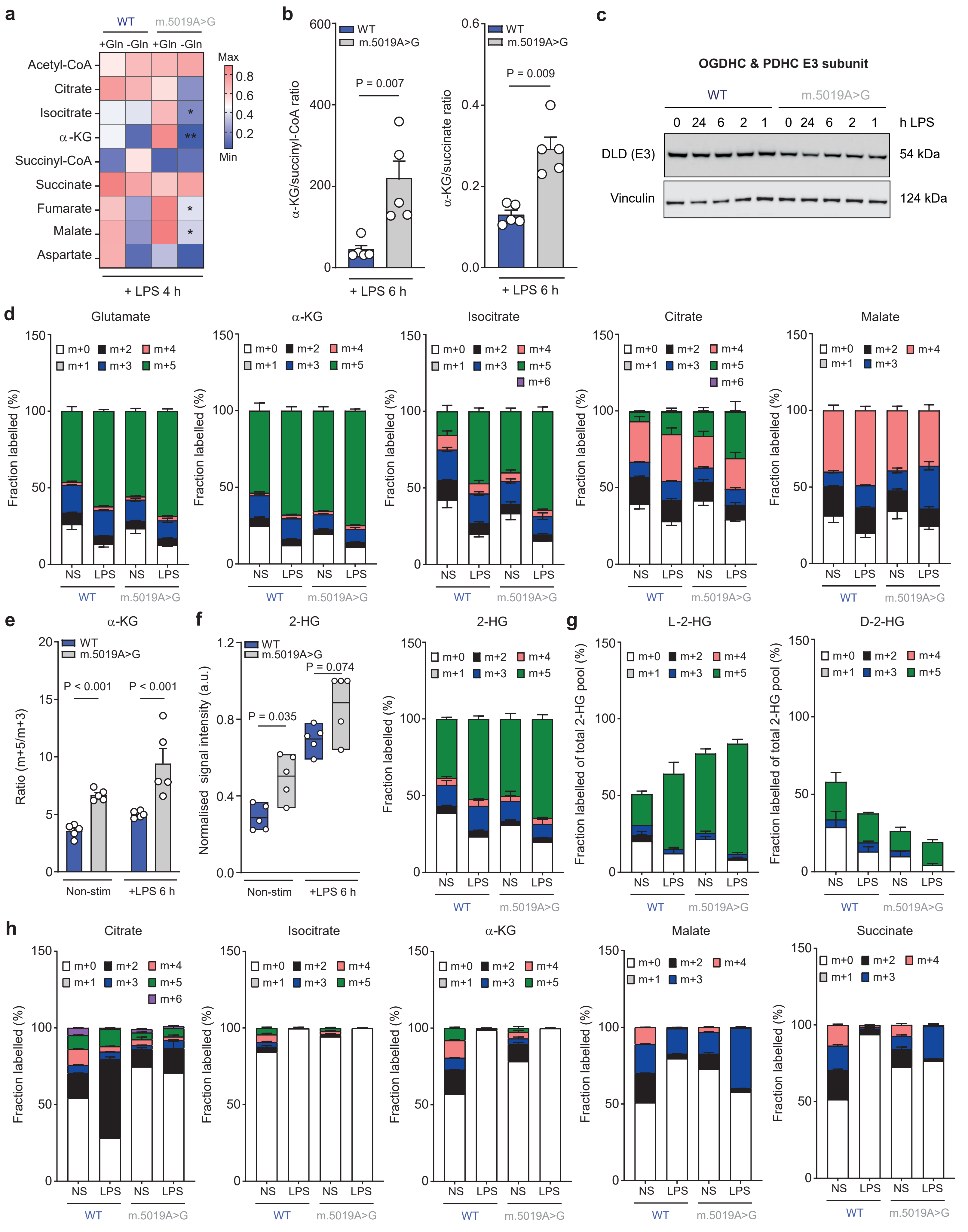

Supplementary figure 4 – TCA cycle remodelling and U-^{13}C-glutamine and-glucose tracing in *m.5019A>G* macrophages

a, Heatmap comparing metabolite levels in lipopolysaccharide (LPS)-stimulated wildtype (WT) and *m.5019A>G* BMDMs in the presence or absence of glutamine (Gln) (n = 3; LPS 4 h) (Isocitrate, P = 0.024324; α-KG, P = 0.000802; Fumarate, P = 0.024324; Malate, P = 0.024324). **b**, α-Ketoglutarate (α-KG)/succinyl-CoA and α-KG/succinate ratio in LPS-stimulated WT and *m.5019A>G* BMDMs (n = 5; LPS 6 h). **c,** Oxoglutarate dehydrogenase complex (OGDHC) and pyruvate dehydrogenase complex (PDHC) E3 subunit (DLD) levels from LPS time course analysis in WT and *m.5019A>G* BMDMs (n = 3; LPS 0, 1, 2, 6 & 24 h). Representative blot shown. **d,** Total isotopologue distribution (% fraction labelling) of tricarboxylic acid (TCA) cycle metabolites and glutamate from U-^{13}C-glutamine tracing in non-stimulated (non-stim) and LPS-stimulated WT and *m.5019A>G* BMDMs (n = 5; LPS 6 h). **e**, m+5/m+3 ratio of α-KG in non-stim and LPS-stimulated WT and *m.5019A>G* BMDMs (n = 5; LPS 6 h) (P = 0.005062; P = 0.000578). **f**, 2-hydroxyglutarate (2-HG) levels and m+5 labelling in 2-HG from U-^{13}C-glutamine in non-stim and LPS-stimulated WT and *m.5019A>G* BMDMs (n = 3; LPS 6 h). **g,** Total isotopologue distribution (% fraction labelling) of L-2-HG and D-2-HG from U-^{13}C-glutamine tracing in non-stim and LPS-stimulated WT and *m.5019A>G* BMDMs (n = 5; LPS 6 h). **h,** Total isotopologue distribution (% fraction labelling) of TCA cycle metabolites from U-^{13}C-glucose tracing in non-stim and LPS-stimulated WT and *m.5019A>G* BMDMs (n = 3; LPS 24 h). Data are mean or mean ± s.e.m. n number represents independent biological replicates (mice) from a minimum of three independent experiments. P values calculated using two-tailed Student's t-test for two group comparisons or multiple two-tailed unpaired t-tests corrected for multiple comparisons using Benjamini, Krieger and Yekutieli method. *** $P < 0.001$ ** $P < 0.01$ * $P < 0.05$.

Supplementary figure 5

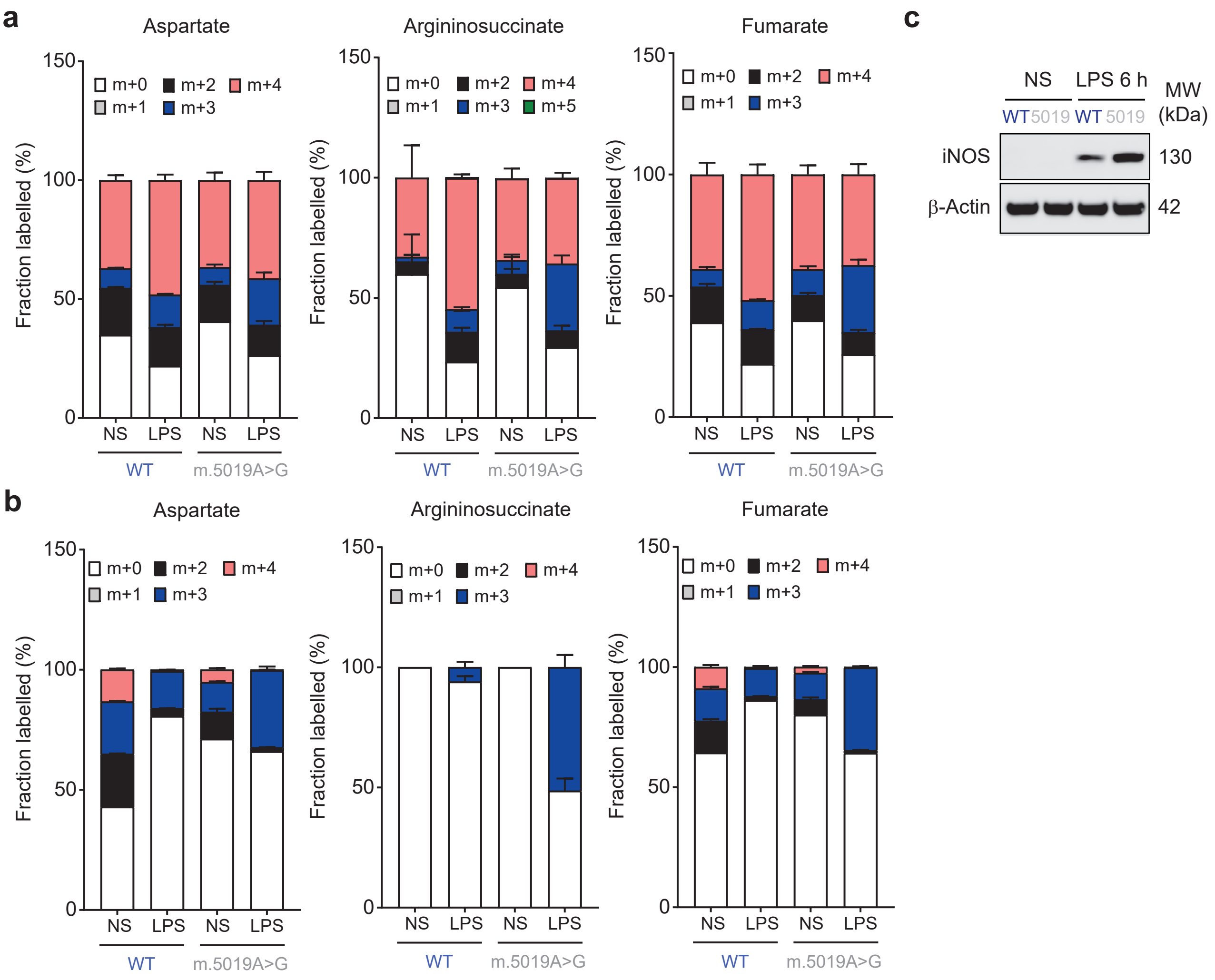

Supplementary figure 5 – U-^{13}C-glutamine and -glucose tracing into the AAS in *m.5019A>G* macrophages

a, Total isotopologue distribution (% fraction labelling) of aspartate, argininosuccinate and fumarate from U-^{13}C-glutamine tracing in non-stimulated (non-stim) and lipopolysaccharide (LPS)-stimulated wildtype (WT) and *m.5019A>G* BMDMs (*n* = 5; LPS 6 h). **b,** Total isotopologue distribution (% fraction labelling) of aspartate, argininosuccinate and fumarate from U-^{13}C-glucose tracing in non-stim and LPS-stimulated WT and *m.5019A>G* BMDMs (*n* = 5; LPS 24 h). **c**, Inducible nitric oxide synthase (iNOS) protein levels in non-stim and LPS-stimulated WT and *m.5019A>G* BMDMs (*n* = 5; LPS 6 h). Representative blot shown. Data are mean ± s.e.m. *n* number represents independent biological replicates (mice) from a minimum of three independent experiments.

Supplementary figure 6

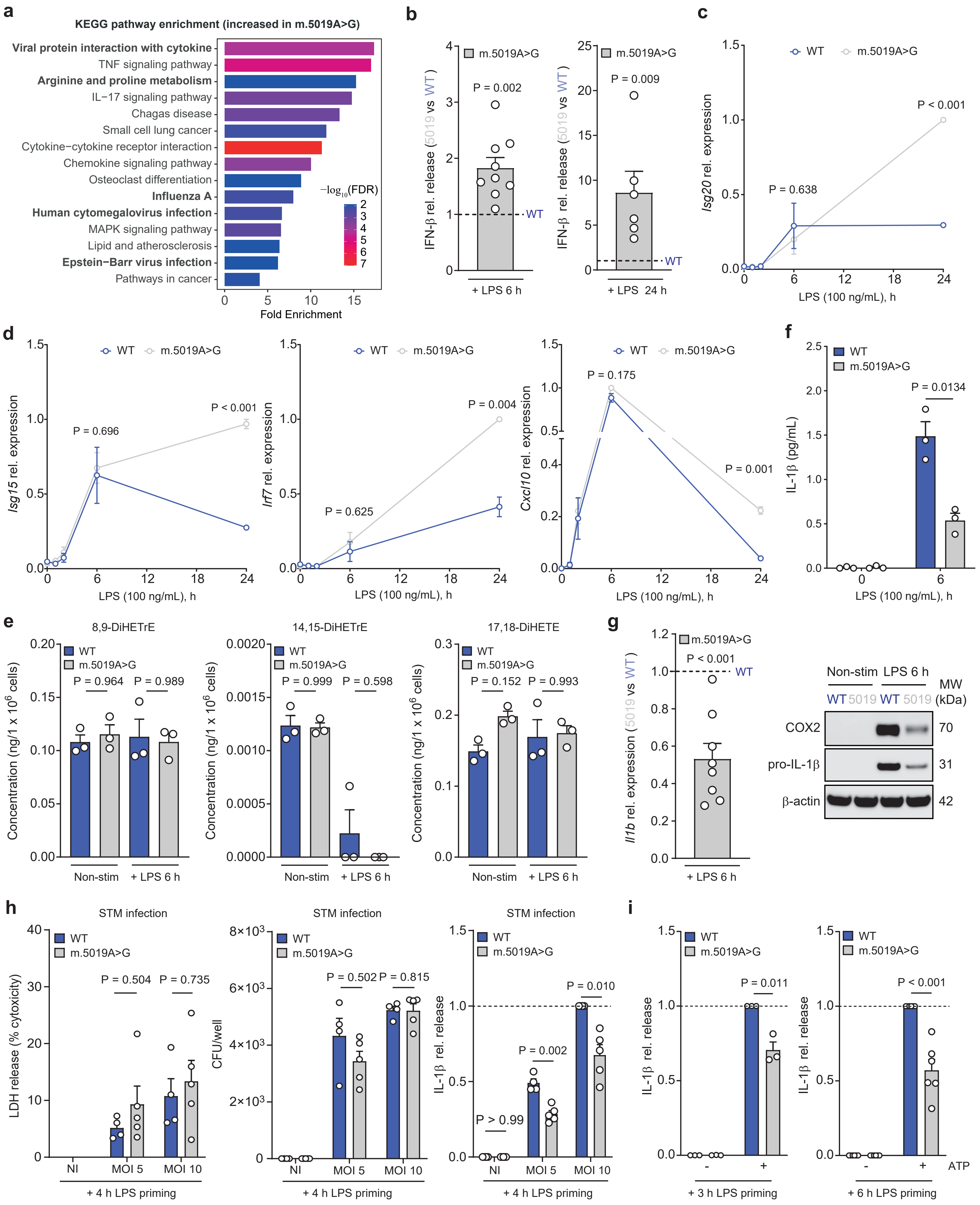

Supplementary figure 6 – Increased type I IFN signalling and reduced IL-1β and COX2 levels in *m.5019A>G* macrophages

a, Overrepresentation analysis (ORA) analysis using KEGG pathway enrichment of increased transcripts in lipopolysaccharide (LPS)-stimulated *m.5019A>G* vs wildtype (WT) BMDMs (n = 3; LPS 1 h). **b**, Interferon-β (IFN-β) release in LPS-stimulated *m.5019A>G* vs WT BMDMs (*n* = 9; LPS 6 h; *n* = 6 LPS 24 h). **c-d,** *Isg20* (*P* = 0.000003), *Isg15* (*P* = 0.000120), *Irf7 and Cxcl10* expression from LPS time course analysis in WT and *m.5019A>G* BMDMs (*n* = 3; LPS 0, 1, 2, 6 & 24 h). **e**, Oxylipin profiling of cell culture medium (CCM) in non-stim and LPS-stimulated WT and *m.5019A>G* BMDMs (*n* = 3; LPS 6 h). **f**, Interleukin-1β (IL-1β) quantification from Olink target T48 mouse cytokine and chemokine profiling of CCM in non-stim and LPS-stimulated *m.5019A>G* vs WT BMDMs (*n* = 3; LPS 6 h). **g,** *Il1b* expression (*n* = 8; *P* = 0.000063) and cyclooxygenase 2 (COX2) and pro-IL-1β protein levels (*n* = 5) in non-stim and LPS-stimulated WT and *m.5019A>G* BMDMs (LPS 6 h). Representative blot shown. **h**, IL-1β release (30 mins), LDH release (30 mins) and bacterial colony forming units (CFU) (1.5 h) following *Salmonella typhimurium* (STM) infection (multiplicity of infection (MOI) 5 and 10) of LPS-primed (4 h) WT (*n* = 4) and *m.5019A>G* (*n* = 5) BMDMs. **i,** IL-1β release following ATP stimulation of LPS-primed WT and *m.5019A>G* BMDMs (*n* = 3; LPS 3 h and *n* = 6; LPS 6 h) (*P* = 0.000151). Data are mean ± s.e.m. *n* number represents independent biological replicates (mice) from a minimum of three independent experiments. *P* values calculated using two-tailed Student's t-test for two group comparisons or multiple two-tailed unpaired t-tests corrected for multiple comparisons using Benjamini, Krieger and Yekutieli method.

Supplementary figure 7

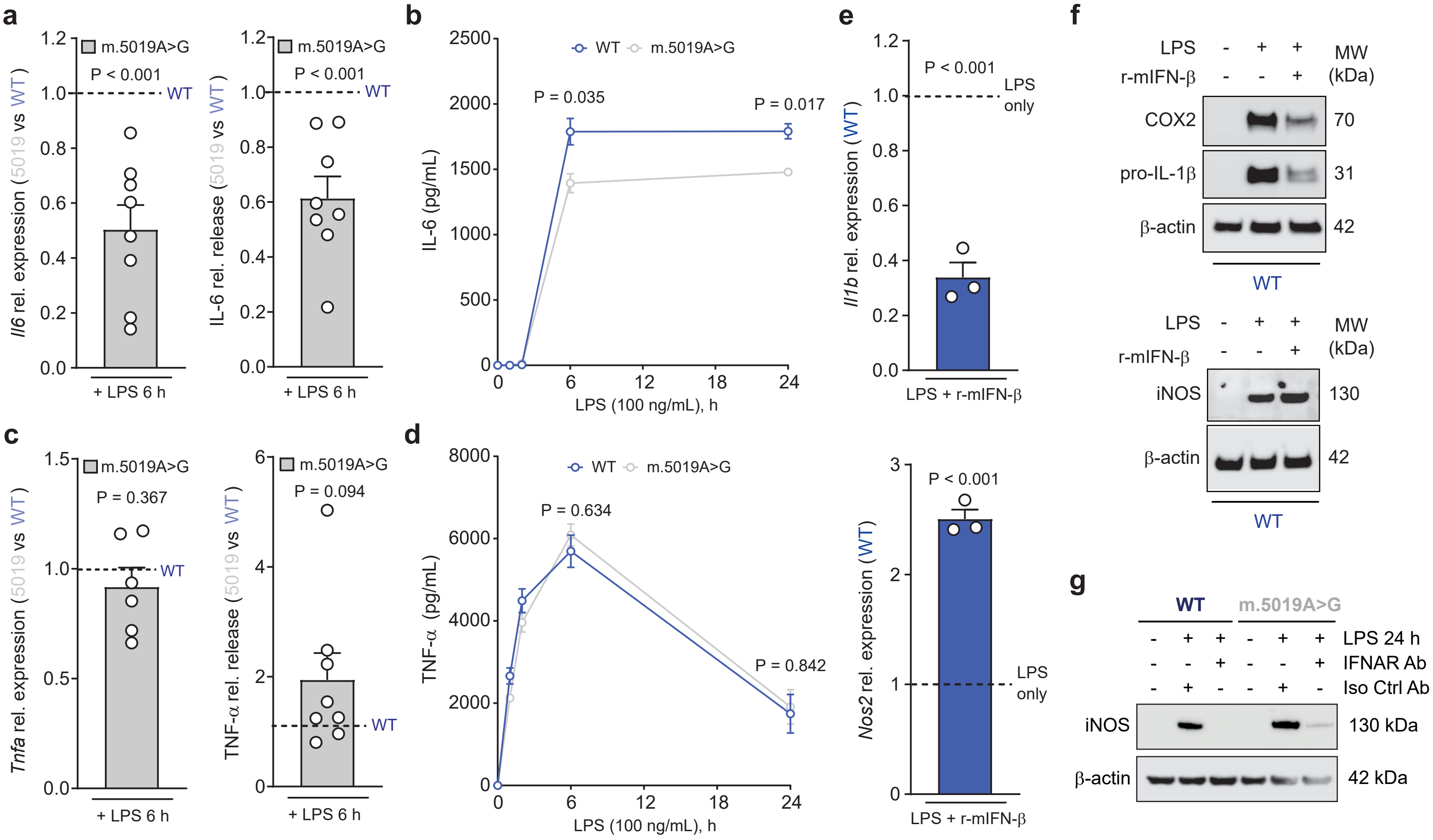

Supplementary figure 7 – Reduced *Il6* expression and IL-6 release in *m.5019A>G* macrophages

a, *Il6* expression (*P* = 0.000072) and interleukin-6 (IL-6) release (*P* = 0.0003) in lipopolysaccharide (LPS)-stimulated *m.5019A>G* vs wildtype (WT) BMDMs (*n* = 8; LPS 6 h). **b,** IL-6 release from LPS time course analysis in WT and *m.5019A>G* BMDMs (*n* = 3; LPS 0, 1, 2, 6 & 24 h). **c,** *Tnfa* expression (*n* = 6) and tumour necrosis factor-α (TNF-α) release (*n* = 8) in LPS-stimulated *m.5019A>G* vs WT BMDMs (LPS 6 h). **d,** TNF-α release from LPS time course analysis in WT and *m.5019A>G* BMDMs (*n* = 3; LPS 0, 1, 2, 6 & 24 h). **e-f**, *Il1b* (*P* = 0.0003) and *Nos2* (*P* = 0.000063) expression and pro-interleukin-1β (IL-1β), cyclooxygenase 2 (COX2) and inducible nitric oxide synthase (iNOS) protein levels in LPS-stimulated and LPS with recombinant mouse (r-m)IFN-β–stimulated WT macrophages (*n* = 3; 6 h). Representative blot shown. **h,** COX2 and pro-IL-1β protein levels in non-stim and LPS-stimulated WT and *m.5019A>G* BMDMs treated with an anti-interferon-α/β receptor (IFNAR) monoclonal antibody (Ab) or isotype control Ab (*n* = 3; LPS 6 h). Representative blot shown. Data are mean ± s.e.m. *n* number represents independent biological replicates (mice) from a minimum of three independent experiments. *P* values calculated using two-tailed Student's t-test for two group comparisons or multiple two-tailed unpaired t-tests corrected for multiple comparisons using Benjamini, Krieger and Yekutieli method.

Supplementary Figure 8

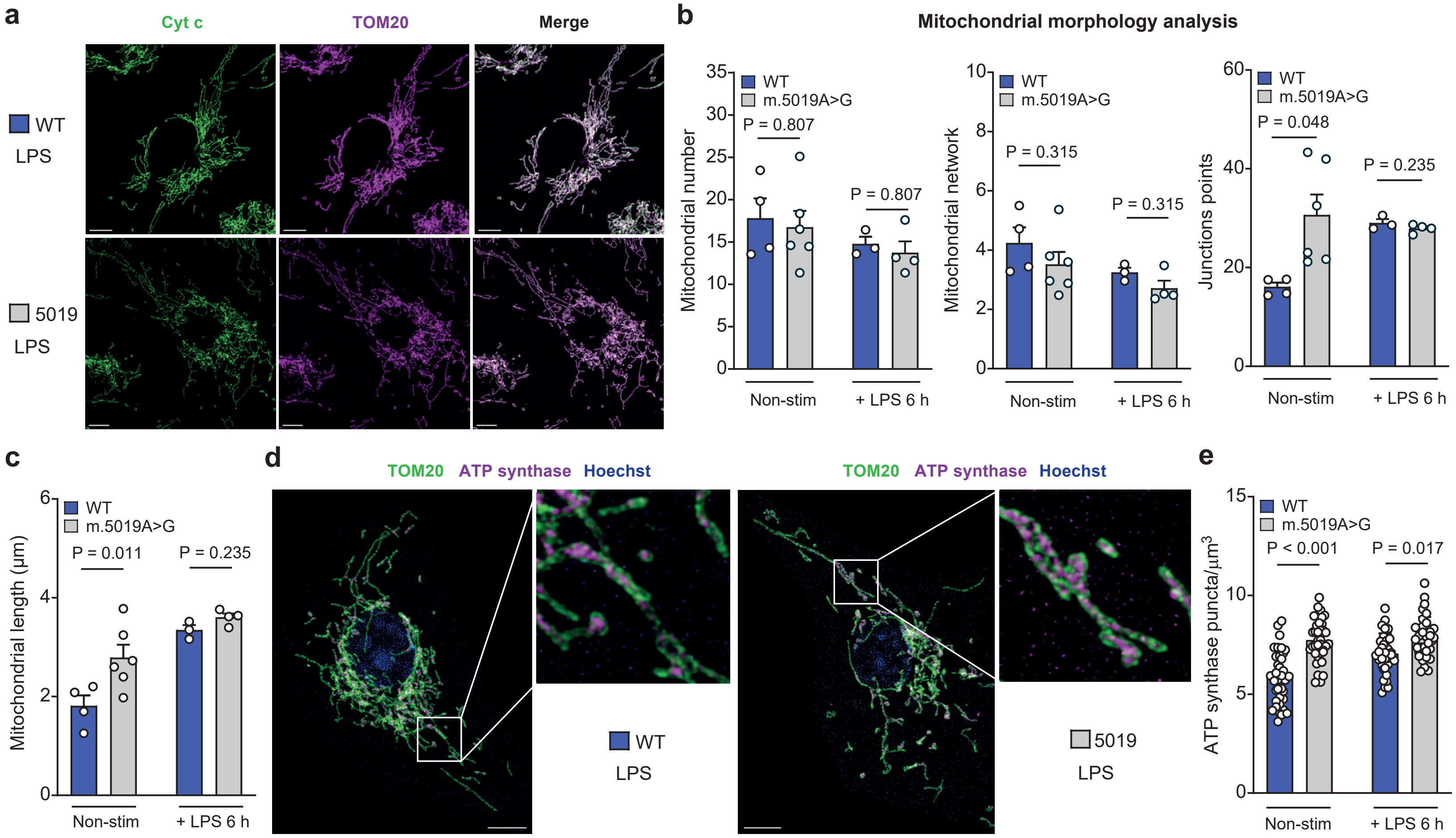

Supplementary figure 8 – Mitochondrial network remodelling in *m.5019A>G* macrophages

a-c, Representative immunofluorescence staining of cytochrome c (Cyt c) and translocase of the outer membrane 20 (TOM20) coupled to confocal microscopy in lipopolysaccharide (LPS)-stimulated wildtype (WT) and *m.5019A>G* BMDMs (**a**) and mitochondrial morphology analysis in non-stimulated (non-stim) WT (*n* = 4), non-stim *m.5019A>G* (*n* = 6), LPS-stimulated WT (*n* = 3), and LPS-stimulated *m.5019A>G* (*n* = 4) BMDMs (**b, c**) (LPS 6 h; minimum of 20 cells analysed per condition per biological replicate). Scale bars: 5 μm. **d-e,** Representative immunofluorescence staining of TOM20 and ATP synthase coupled to super-resolution microscopy in LPS-stimulated WT and *m.5019A>G* BMDMs (**d**) and ATP synthase puncta analysis of non-stim WT (*n* = 3), non-stim *m.5019A>G* (*n* = 3), LPS-stimulated WT (*n* = 2) and LPS-stimulated *m.5019A>G* (*n* = 3) BMDMs (**e**) (LPS 6 h; minimum of 33 cells from independent biological replicates) (*P* = 0.0000001). Scale bars: 5 μm. Data are mean ± s.e.m or ± s.d. *n* number represents independent biological replicates (mice) from a minimum of two independent experiments. *P* values calculated using multiple two-tailed unpaired t-tests corrected for multiple comparisons using Holm-Sidak method or one-way ANOVA corrected for multiple comparisons using the Kruskal-Wallis method.

Supplementary figure 9

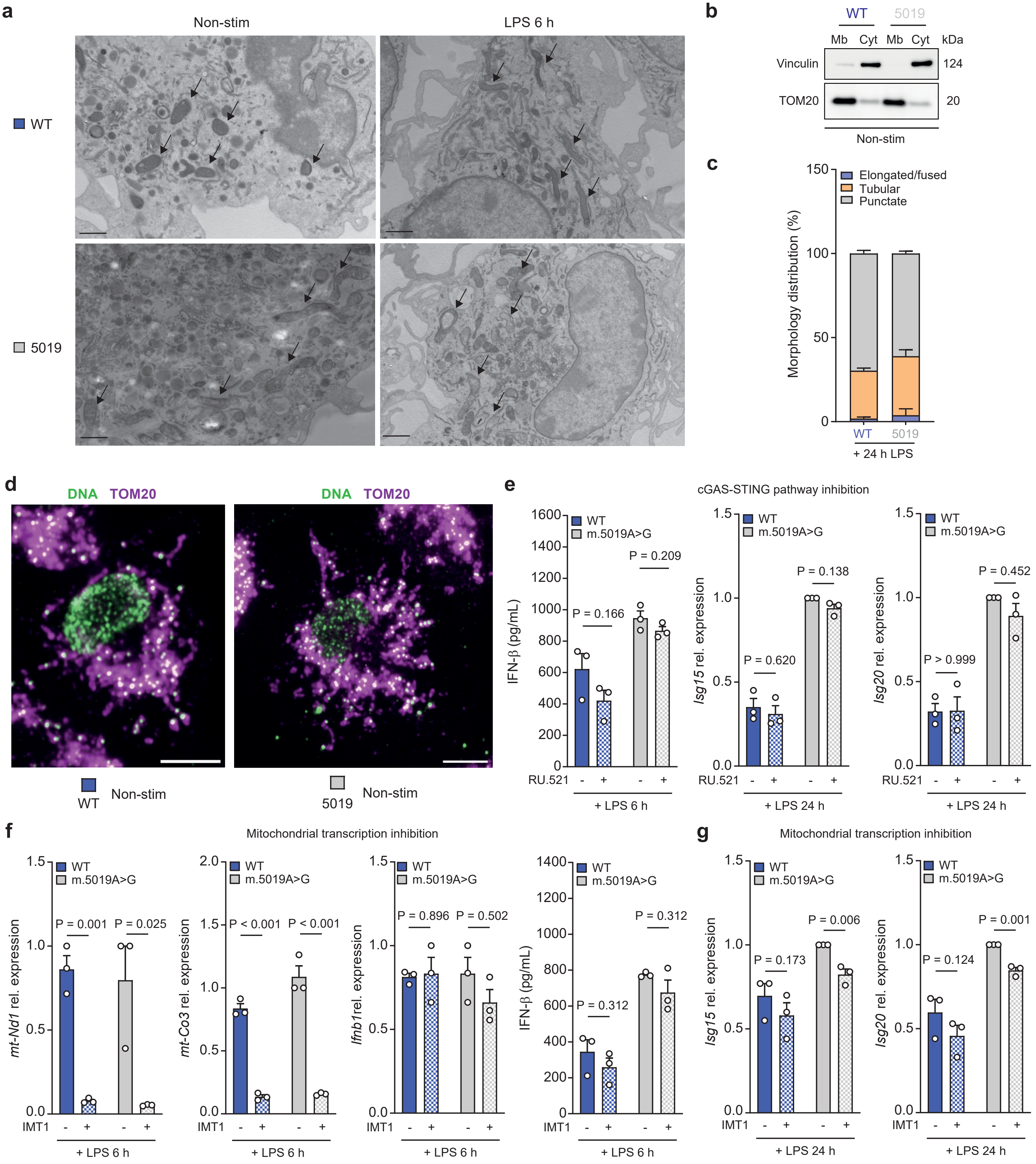

Supplementary figure 9 – Early phase type I IFN in *m.5019A>G* macrophages is independent of mitochondrial nucleic acids

a, Representative transmission electron microscope (TEM) images of non-stimulated (non-stim) and lipopolysaccharide (LPS)-stimulated WT and *m.5019A>G* BMDMs (*n* = 3; LPS 6 h; mitochondria from a minimum of 9 cells were analysed per condition per biological replicate). Scale bars: 0.5 μm. Black arrows indicate mitochondria. **b**, Vinculin and translocase of the outer membrane 20 (TOM20) levels in membrane (Mb) and cytosolic (Cyt) fraction of non-stim WT and *m.5019A>G* BMDMs (*n* = 3). Representative blot shown. **c,** Mitochondrial morphology distribution in LPS-stimulated WT and *m.5019A>G* BMDMs (*n* = 3; LPS 24 h; minimum of 20 cells analysed per condition per biological replicate). **d,** Representative immunofluorescence staining of DNA and TOM20 coupled to confocal microscopy in non-stim WT and *m.5019A>G* BMDMs (*n* = 3; LPS 24 h; minimum of 20 cells analysed per condition per biological replicate). **e,** Interferon-β (IFN-β) release and *Isg15* and *Isg20* expression in LPS-stimulated WT and *m.5019A>G* macrophages pre-treated with cyclic GMP-AMP synthase (cGAS) inhibitor RU.521 or vehicle control (DMSO) for 1 h (*n* = 3; LPS 6 h). **f,** *mt-Nd1*, *mt-Co3* (*P* = 0.000158; *P* = 0.000470), *Ifnb1* expression and IFN-β release in LPS-stimulated WT and *m.5019A>G* macrophages pre-treated with inhibitor of mitochondrial transcription 1 (IMT1) or vehicle control (DMSO) for 24 h (*n* = 3; LPS 6 h). **g,** *Isg15 and Isg20* expression in LPS-stimulated WT and *m.5019A>G* macrophages pre-treated with IMT1 or vehicle control (DMSO) for 24 h (*n* = 3; LPS 24 h). Data are mean ± s.e.m. *n* number represents independent biological replicates (mice) from a minimum of three independent experiments. *P* values calculated using multiple two-tailed unpaired t-tests corrected for multiple comparisons using Benjamini, Krieger and Yekutieli method.

Supplementary figure 10

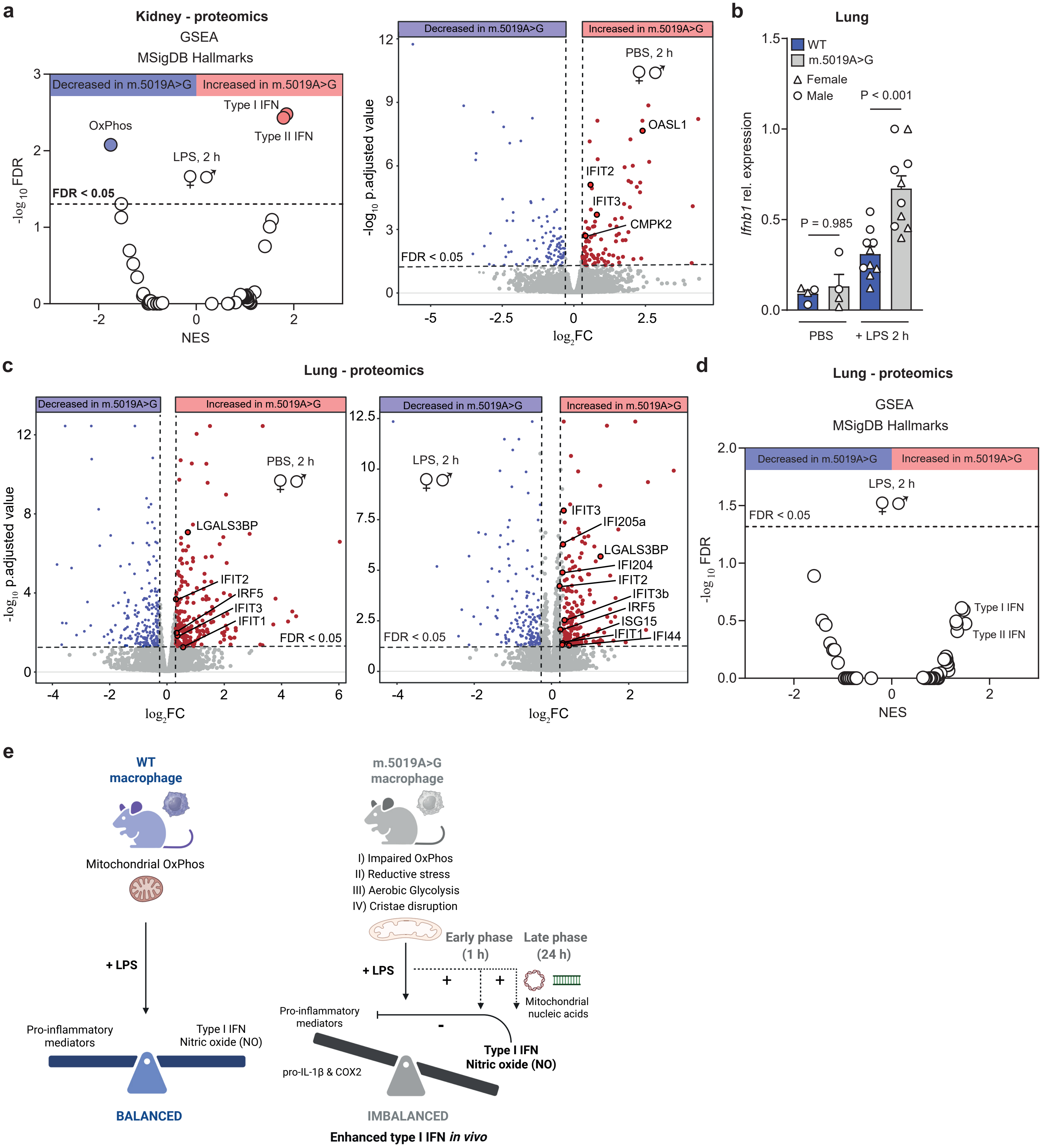

Supplementary figure 10 – Elevated type I IFN levels in *m.5019A>G* mice

a, Gene set enrichment analysis (GSEA) analysis of kidney tissue proteomic analysis of *m.5019A>G* mice vs wildtype (WT) mice injected i.p. with lipopolysaccharide (LPS) (*n* = 10; 2 h; left) and volcano plot of kidney tissue proteomic analysis of *m.5019A>G* mice vs WT mice injected i.p. with phosphate buffered saline (PBS) (*n* = 4; 2 h; right). **b,** *Ifnb1* expression in lung tissue of *m.5019A>G* mice vs WT mice serum injected i.p. with PBS (*n* = 4; 2 h) or LPS (*n* = 10; 2 h) (*P* = 0.000301). **c,** Volcano plot of lung tissue proteomic analysis of *m.5019A>G* mice vs WT mice injected i.p. with PBS (*n* = 4; 2 h; left) or LPS (*n* = 10; 2 h; right). **d,** GSEA analysis of lung tissue proteomic analysis of *m.5019A>G* mice vs WT mice injected i.p. with LPS (*n* = 10; 2 h). **e,** Summary of findings comparing WT and *m.5019A>G* macrophages and mice. Data are $\log_2$FC or mean ± s.e.m. *n* number represents independent biological replicates (mice) from a minimum of two independent experiments. *P* values calculated using multiple two-tailed unpaired t-tests corrected for multiple comparisons using Benjamini, Krieger and Yekutieli method. **e**, Created in BioRender. Dwane, L. (2025) https://BioRender.com/d1gscu7.

Supplementary tables

Supplementary Table 1 – Pyrosequencing primers

m.5019A>G PCR F	5'-ATATACTAGTCCGCGAGCCTTC-3'
m.5019A>G PCR R	5'-[Biotin]-GCAAATTCGAAGGTGTAGAGAAA-3'
Sequencing primer	5'-CACACAAGTTTAACTTCTGA-3'

Supplementary Table 2 – ddPCR primers

Gene	Sequence
mt-Nd1 forward	5'-GAGCCTCAAACTCCAAATACTCACT-3'
mt-Nd1 reverse	5'-GAACTGATAAAAGGATAATAGCTATGGTTACTTCA-3'
ActB forward	5'-CTGCTCTTTCCCAGACGAGG-3'
ActB reverse	5'-AAGGCCACTTATCACCAGCC-3'
ActB/FAM/BHQ1	5'6-FAM/ ATTGCCTTTCTGACTAGGTG /3BHQ_1
mt-Nd1/HEX/BHQ1	5'HEX/ CCGTAGCCCAAACAAT /3BHQ_1

Supplementary Table 3 – Antibodies used for all methods

Antibody	Dilution	Source	Identifier
Pro-IL-1β	1/1000	Cell Signalling Technology	12507; RRID: AB_2721117
COX2	1/1000	Cell Signalling Technology	12282S; RRID: AB_2571729
iNOS	1/1000	Cell Signalling Technology	13120S; RRID: AB_2687529
ISG15	1/1000	Cell Signalling Technology	89771S
IRF7	1/1000	Cell Signalling Technology	72073S; RRID: AB_3073735
IRF3	1/1000	Cell Signalling Technology	4302S
Phospho-IRF3	1/1000	Cell Signalling Technology	4947S; RRID: AB_823547
Total OxPhos	1/250	Abcam	110413; RRID: AB_2629281
DLD	1/1000	Abcam	ab133551
β-actin	1/1000	Cell Signalling Technology	4970; RRID: AB_2223172
Cytochrome c	1/1000	BD Biosciences	556432; RRID: AB_396416
DRP1	1/1000	BD Biosciences	611113; RRID: AB_398424
TOM20	1/1000	Proteintech	11802-1-AP; RRID: AB_2207530
TOM20	1/500	Abcam	ab232589; RRID: AB_3065091
ATP synthase	1/500	Merck	MAB3494; RRID: AB_177597
DNA	1/1000	Merck	CBL186; RRID: AB_11213573
Vinculin	1/1000	Cell Signalling Technology	13901; RRID: AB_2728768
F4/80	1/100	ThermoFisher scientific	12-4801-80; 12-4801-80; RRID: AB_465922
Anti-rabbit IgG, HRP-linked	1/2000	Cell Signalling Technology	7074; RRID: AB_2099233
Anti-mouse IgG, HRP-linked	1/2000	Cell Signalling Technology	7076; RRID: AB_330924
Alexa Fluor 488, Anti-mouse IgG1	1/1000	ThermoFisher scientific	A21121; RRID: AB_2535764
Alexa Fluor 568, Anti-rabbit IgG	1/1000	ThermoFisher scientific	A11036; RRID: AB_10563566

Supplementary Table 4 – RT-qPCR primers

**Primer**		**Sequence**
Il1b	Forward	5’-TGCCACCTTTTGACAGTGATG-3’
	Reverse	5’-TGATGTGCTGCTCGCAGATT-3’
Il6	Forward	5’-TGAGAAAAGAGTTGTGCAATGG-3’
	Reverse	5’-GGTACTCCAGAAGACCAGAGG-3’
Tnfa	Forward	5’-GATCGGTCCCCAAAGGGATG-3’
	Reverse	5’-TGAGAAGATGATCTGAGTGTGAG-3’
Rps18	Forward	5’-GCCGCCATGTCTCTAGTGA -3’
	Reverse	5’-ATGAGCATATCTCCGCCCCA-3’
mt-Co3	Forward	5’-CCTCGTACCAACACATGATCTAGG-3’
	Reverse	5’-AGTGGGACTTCTAGAGGGTTAAGTG-3’
mt-Nd1	Forward	5’-GAGCCTCAAACTCCAAATACTCACT-3’
	Reverse	5’-GAACTGATAAAAGGATAATAGCTATGGTTA -3’
Ifnb1	Forward	5’-AACTCCACCAGCAGACAGTG-3’
	Reverse	5’-GGTACCTTTGCACCCTCCAG-3’
Isg15	Forward	5’-TCTGACTGTGAGAGCAAGCAG-3’
	Reverse	5’-ACCTTTAGGTCCCAGGCCATT-3’
Isg20	Forward	5’-GGCACTGAGACAGGGCTTT-3’
	Reverse	5’-GAGGCCACTCACCCTTTGAG-3’
Irf7	Forward	5’-GAATCCGAGTCTGGGGCAG-3’
	Reverse	5’-GCTGCGCTCGGTGAG-3’
Cxcl10	Forward	5’-TCACTCCAGTTAAGGAGCCC-3’
	Reverse	5’-CCACGTGTTGAGATCATTGCC-3’
Nos2	Forward	5’-AAACCCCTTGTGCTGTTCTC-3’
	Reverse	5’-GGGATTCTGGAACATTCTGTGC-3’
