# Supplementary material for: An inherited mitochondrial DNA mutation remodels inflammatory cytokine responses in macrophages and in vivo in mice
Source: Nat Commun. 2025 Nov 20;16:10222. doi: 10.1038/s41467-025-65023-4 (PMC12635290; doi:10.1038/s41467-025-65023-4)
Supplement: Supplementary file 2 — Reporting Summary [file 41467_2025_65023_MOESM2_ESM.pdf]

nature portfolio

Corresponding author(s): Dylan G. Ryan

Last updated by author(s): 10/09/2025

Reporting Summary

Nature Portfolio wishes to improve the reproducibility of the work that we publish. This form provides structure for consistency and transparency in reporting. For further information on Nature Portfolio policies, see our Editorial Policies and the Editorial Policy Checklist.

Please do not complete any field with "not applicable" or n/a. Refer to the help text for what text to use if an item is not relevant to your study.
For final submission: please carefully check your responses for accuracy; you will not be able to make changes later.

Statistics

For all statistical analyses, confirm that the following items are present in the figure legend, table legend, main text, or Methods section.

n/a	Confirmed	
☐	☒	The exact sample size (*n*) for each experimental group/condition, given as a discrete number and unit of measurement
☐	☒	A statement on whether measurements were taken from distinct samples or whether the same sample was measured repeatedly
☐	☒	The statistical test(s) used AND whether they are one- or two-sided<br>*Only common tests should be described solely by name; describe more complex techniques in the Methods section.*
☒	☐	A description of all covariates tested
☐	☒	A description of any assumptions or corrections, such as tests of normality and adjustment for multiple comparisons
☐	☒	A full description of the statistical parameters including central tendency (e.g. means) or other basic estimates (e.g. regression coefficient) AND variation (e.g. standard deviation) or associated estimates of uncertainty (e.g. confidence intervals)
☐	☒	For null hypothesis testing, the test statistic (e.g. *F*, *t*, *r*) with confidence intervals, effect sizes, degrees of freedom and *P* value noted<br>*Give P values as exact values whenever suitable.*
☒	☐	For Bayesian analysis, information on the choice of priors and Markov chain Monte Carlo settings
☒	☐	For hierarchical and complex designs, identification of the appropriate level for tests and full reporting of outcomes
☐	☒	Estimates of effect sizes (e.g. Cohen's *d*, Pearson's *r*), indicating how they were calculated

Our web collection on statistics for biologists contains articles on many of the points above.

Software and code

Policy information about availability of computer code

Data collection	Raw files for proteomics were analysed and quantified by searching against the Uniprot Mus Musculus data base using DIA-NN 1.8 (https://github.com/vdemichev/DiaNN). LC-MS data for metabolomics were analysed with a targeted approach using ThermoFisher Scientific Xcalibur. Samples were quantified using MassLynx 4.1 software to determine the peak of area for UQ9 and UQ9H2. Peak area integration for ATP, ADP and AMP was performed using LabSolutions software (Shimadzu).Chromatographic peaks for oxylipins were integrated using Sciex OS 3.3.0 software (Sciex).
Data analysis	Graphpad Prism 10.4.2 was used to calculate statistics in plots using appropriate statistical tests depending on the data including two-tailed unpaired t test, one-way ANOVA and multiple t tests. Flow cytometry analysis was performed on a BD Fortessa flow cytometer and further analysed using FlowJo software version 10.10.0 (FlowJo LLC). Heteroplasmy was then called using Pyromark® analysis software (Qiagen) and reported as the percentage of mutant base present in the sample. ddPCR results were analysed using QuantaSoft analysis software (Bio-Rad) and the average mtDNA copy number (HEX probe) was normalised to the nuclear DNA copy number (FAM probe). All Seahorse XF24 analyser results were acquired with Wave software (Agilent) and analysed with Seahorse XF test report generators. For Oroboros experiments, oxygen flux (JO2), which is directly proportional to OCR, was continuously recorded with a 2 sec sampling rate using DatLab software 6.1 (Oroboros Instruments, Austria). All confocal images were acquired using a Zyla 4.2 PLUS sCMOS camera attached to an Andor DragonFly 500 confocal spinning disk mounted on a Nikon Eclipse TiE microscope using a CFI Plan Apochromat lambda 100X oil immersion objective and using the Fusion user interface (Andor). 7-stacks of 0.2 um were acquired using appropriate lasers. All images from the same experiment were acquired with the same parameters including exposure time and laser intensities. Images were compiled by "max projection" and analysed with Fiji ImageJ (NIH). Fixed cell super-resolution images for analysis of mitochondrial morphology in 3D were obtained with the Zeiss Elyra7 lattice SIM, using the Plan-Apochromat 63x/1.4 Oil DIC M27 objective with 15 phases and 0.091 µm intervals. Images were acquired with 20 ms exposure time with 405 nm (20.0%), 488 nm (4.0%), and 561 nm (6.0%) lasers. Standard deconvolution was performed in Zen Black. Using Imaris 10.1.0, objects in separate channels were segmented and rendered in 3D with the Surfaces function. Transmission electron

microscopy. (TEM) images were acquired using an ORCA HR high resolution CCD camera (Advanced Microscopy Techniques Corp, Danvers USA). Western blot membranes were imaged using an Amersham Imager 680 and the SignalFire Plus ECL Reagents (6883, Cell Signalling). Blots were subsequently analysed using Image Lab software (Bio-Rad). For proteomics, protein-wise linear models combined with empirical Bayes statistics were used for the differential expression analyses. For proteomics, the Bioconductor package limma was used to carry out the analysis using an R based online tool. For RNA sequencing, differential gene expression analysis was done using the counted reads and the R package edgeR version 3.26.5 (R version 3.6.1) for the pairwise comparisons.

For manuscripts utilizing custom algorithms or software that are central to the research but not yet described in published literature, software must be made available to editors and reviewers. We strongly encourage code deposition in a community repository (e.g. GitHub). See the Nature Portfolio guidelines for submitting code & software for further information.

Data

Policy information about availability of data

All manuscripts must include a data availability statement. This statement should provide the following information, where applicable:

- Accession codes, unique identifiers, or web links for publicly available datasets
- A description of any restrictions on data availability
- For clinical datasets or third party data, please ensure that the statement adheres to our policy

All source data supporting the findings of this study are provided in the Source Data file. The raw and processed RNA-sequencing datasets generated in this study have been deposited in DRYAD (https://doi.org/10.5061/dryad.ksn02v7fn) and ArrayExpress (E-MTAB-15591). All raw proteomics datasets have been deposited in PRIDE (Project accession code: PXD060284 [https:// proteomecentral.proteomexchange.org/cgi/GetDataset?ID=PXD060284]). All raw lipidomics data has been deposited in the Cardiff University Research Data repository (https://doi.org/10.17035/cardiff.30074749) accompanied by Oxylipin sMRM method report (https://doi.org/10.5281/zenodo.17078883). All raw metabolomics datasets have been deposited in MassIVE under accession code MSV000099126 (https://doi.org/10.25345/C58C9RH3G).

Research involving human participants, their data, or biological material

Policy information about studies with human participants or human data. See also policy information about sex, gender (identity/presentation), and sexual orientation and race, ethnicity and racism.

Reporting on sex and gender	N/A
Reporting on race, ethnicity, or other socially relevant groupings	N/A
Population characteristics	N/A
Recruitment	N/A
Ethics oversight	N/A

Note that full information on the approval of the study protocol must also be provided in the manuscript.

Field-specific reporting

Please select the one below that is the best fit for your research. If you are not sure, read the appropriate sections before making your selection.

☒ Life sciences ☐ Behavioural & social sciences ☐ Ecological, evolutionary & environmental sciences

For a reference copy of the document with all sections, see nature.com/documents/nr-reporting-summary-flat.pdf

Life sciences study design

All studies must disclose on these points even when the disclosure is negative.

Sample size	No statistical methods were used to predetermine sample sizes. The number of replicates were decided based on previous published data in the field that yields statistically interpretable data, while adhering to the 3Rs when using primary bone marrow-derived macrophages (BMDMs) and mice. A minimum of 3 independent biological replicates (n) (mice) were used per experiment in a minimum of 2 independent batches to account for biological variability, with the exception of super resolution microscopy where the n number was each individual cell (33-38 cells) from 2-3 independent biological replicates. N number of independent biological replicates (mice) ranged from 3 to 10 per mouse per condition and were performed in multiple experiments ranging from 2-5 batches.
Data exclusions	Data were not excluded from the study.
Replication	The number of biological replicates are included in all the figure legends. All findings in the manuscript were supported and validated using several orthogonal methodological approaches to enhance reproducibility and confidence in reported findings. For example, key RNA sequencing results were validated with quantitative PCR. Key proteomic results were validated with ELISA and Western blot assays. Key metabolic findings were validated with extracellular flux analysis, oxygen flux, steady state LC-MS and stable isotope-assisted tracing.

Mitochondrial morphology analysis was determined using confocal microscopy, super resolution microscopy and transmission electron microscopy.

Randomization

All m.5019A>G BMDMs and mice were age- and sex-matched to their wildtype control counterparts. BMDMs from individual mice were randomly divided into groups for treatment. Mice were randomly assigned to control or treatment group.

Blinding

Blinding was not used for in vitro BMDM experiments for practical reasons and not readily possible due to observable distinctions in cell behaviour between m.5019A>G and WT macrophages. Blinding was not used for in vivo treatments due to a limited number of investigators. However, the in vivo sepsis score for the movement behaviour, flight reaction and body position were assessed and assigned a score in a blinded manner from video recordings.

Reporting for specific materials, systems and methods

We require information from authors about some types of materials, experimental systems and methods used in many studies. Here, indicate whether each material, system or method listed is relevant to your study. If you are not sure if a list item applies to your research, read the appropriate section before selecting a response.

Materials & experimental systems

n/a	Involved in the study	
☐	☒	Antibodies
☒	☐	Eukaryotic cell lines
☒	☐	Palaeontology and archaeology
☐	☒	Animals and other organisms
☒	☐	Clinical data
☒	☐	Dual use research of concern
☒	☐	Plants

Methods

n/a	Involved in the study	
☒	☐	ChIP-seq
☐	☒	Flow cytometry
☒	☐	MRI-based neuroimaging

Antibodies

Antibodies used

Pro-IL-1beta-1/1000-Cell Signalling Technology -12507; RRID: AB_2721117
COX2 -1/1000-Cell Signalling Technology-12282S; RRID: AB_2571729
iNOS-1/1000-Cell Signalling Technology-13120S; RRID: AB_2687529
ISG15-1/1000 -Cell Signalling Technology-89771S
IRF7-1/1000-Cell Signalling Technology-72073S; RRID: AB_3073735
IRF3-1/1000-Cell Signalling Technology-4302S
Phospho-IRF3-1/1000-Cell Signalling Technology -4947S; RRID: AB_823547
Total OxPhos -1/250-Abcam -110413; RRID: AB_2629281
DLD-1/1000-Abcam-ab133551
Beta-actin-1/1000-Cell Signalling Technology-4970; RRID: AB_2223172
Cytochrome c-1/1000-BD Biosciences-556432; RRID: AB_396416
DRP1-1/1000-BD Biosciences-611113; RRID: AB_398424
TOM20-1/1000-Proteintech-11802-1-AP; RRID: AB_2207530
TOM20-1/500 Abcam-ab232589; RRID: AB_3065091
ATP synthase-1/500-Merck MAB3494; RRID: AB_177597
DNA-1/1000-Merck-CBL186; RRID: AB_11213573
Vinculin-1/1000-Cell Signalling Technology-13901; RRID: AB_2728768
F4/80-1/100-ThermoFisher scientific-12-4801-80; 12-4801-80; RRID: AB_465922
Anti-rabbit IgG, HRP-linked-1/2000-Cell Signalling Technology-7074; RRID: AB_2099233
Anti-mouse IgG, HRP-linked -1/2000-Cell Signalling Technology-7076; RRID: AB_330924
Alexa Fluor 488, Anti-mouse IgG1-1/1000-ThermoFisher scientific-A21121; RRID: AB_2535764
Alexa Fluor 568, Anti-rabbit IgG-1/1000-ThermoFisher scientific -A11036; RRID: AB_10563566

Validation

All antibodies were used according to manufacturer's instructions. All antibodies were used according to the manufacturer's instructions. Only antibodies that have been validated by the manufacturer were used in this study.

Antibody validation on manufacturer's website is as follows:
Pro-IL-1β (12507): by western blot analysis of extracts from RAW264.7 cells. The expected band appeared at the expected molecular weight.
COX2 (12282S): by western blot analysis of extracts from RAW264.7 cells. The expected band appeared at the expected molecular weight.
iNOS (13120S): by western blot analysis of extracts from RAW264.7 cells. The expected band appeared at the expected molecular weight.
ISG15 (89771S): Western blot analysis of extracts from NIH/3T3, RAW 264.7, and mIMCD-3 cells. The expected band appeared at the expected molecular weight.
IRF7 (72073S): Western blot analysis of extracts from A20 and YB2/0 cells. The expected bands appeared at the expected molecular weights.
IRF3 (4302S): Western blot analysis of extracts from control HeLa cells or IRF-3 knockout HeLa cells. The absence of signal in the IRF-3

knockout HeLa cells confirm specificity of the antibody for IRF-3.The expected band appeared at the expected molecular weight.
Phospho-IRF3 (4947S): Western blot analysis of extracts from HT29 and THP1 cells. The expected band appeared at the expected molecular weight.
Total OxPhos cocktail (ab110413): Western blot analysis of rat liver mitochondria, white adipose tissue, mouse embryonic fibroblasts, mouse brain, human heart mitochondria and human skeletal muscle. The expected bands appeared at the expected molecular weights.
DLD (ab133551) - Western blot analysis was of wild-type and DLD-knockout HeLa cells. The absence of signal in the DLD knockout HeLa cells confirms specificity of the antibody for DLD. The expected band appeared at the expected molecular weight.
Beta-actin (4970) - Western blot analysis of cell extracts from various cell lines. The expected band appeared at the expected molecular weight.
Cytochrome c (556432) - Western blot of mouse lymphoma, HeLa human carcinoma, Jurkat T leukemia, and NIH/3T3 mouse fibroblast cell lysates and immunofluorescence of HeLa human carcinoma. The expected mitochondrial network was observed and overlapped with TOM20.
DRP1 (611113) - Western blot analysis of HCT-8 cell lysate (Human colorectal adenocarcinoma; ATCC CCL-244) and immunofluorescence staining on A431 cells (Human epithelial carcinoma; ATCC CRL-1555).
TOM20 (ab232589) - Immunofluorescence and immunocytochemistry in HeLa cells and mouse small intestine. The expected mitochondrial network was observed and overlapped with Cyt c.
TOM20 (11802-1-AP) - Western blot and immunofluorescence in various cell lines, including HUVEC, HepG2, C6 and HEK-293, and knockdown validated in HEK-193 cells. The expected mitochondrial network was observed.
ATP Synthase (MAB3494) - Immunocytochemistry and immunoprecipitation in rat neurons and glia, and mitochondrial localisation was confirmed.
DNA (CBL186) - has been validated in Immunofluorescence and immunocytochemistry.
Vinculin (13901) - Western blot analysis of extracts from several cell lines. The expected band appeared at the expected molecular weight.
F4/80 (12-4801-80; 12-4801-80) - Staining of C57Bl/6 resident peritoneal exudate cells for flow cytometry.

Animals and other research organisms

Policy information about studies involving animals; ARRIVE guidelines recommended for reporting animal research, and Sex and Gender in Research

Laboratory animals	All mice used in this study were on the C57BL/6J background. Wildtype (WT) mice were purchased from Charles River Laboratories, UK. The m.5019A>G mouse strain (Allele symbol: mt-Tam2Jbst, MGI ID: 6860509) was generously provided from the colony of Patrick F. Chinnery. The mice were provided through an MTA with James. B Stewart. WT and m.5019A>G mice were age and sex matched for all experiments. BMDMs were derived from male and female mice aged 8-33 weeks old. Both male and female WT and m.5019A>G mice (8-12 weeks old) were used for in vivo experiments.
Wild animals	This study did not involve wild animals.
Reporting on sex	Both male and female mice were used for bone marrow-derived macrophage (BMDM) generation and in vivo experiments.
Field-collected samples	This study did not involve field-collected samples.
Ethics oversight	All mouse experiments and breeding were carried out in accordance with the UK Animals (Scientific Procedures) Act, 1986 (Home Office PPL no. PP1740969) and EU Directive 2010/63/EU. All experiments followed ARRIVE 2.0 guidelines and procedures were approved by the University of Cambridge Animal Welfare and Ethical Review Body (AWERB) Committee.

Note that full information on the approval of the study protocol must also be provided in the manuscript.

Plants

Seed stocks	N/A
Novel plant genotypes	N/A
Authentication	N/A

Flow Cytometry

Plots

Confirm that:

☒ The axis labels state the marker and fluorochrome used (e.g. CD4-FITC).

☒ The axis scales are clearly visible. Include numbers along axes only for bottom left plot of group (a 'group' is an analysis of identical markers).

☒ All plots are contour plots with outliers or pseudocolor plots.

☒ A numerical value for number of cells or percentage (with statistics) is provided.

Methodology

Sample preparation	BMDMs were plated at 1 x 10^6 cells/well in 6-well cell culture plates (2 mL total volume) and left to adhere overnight at 37C in a de-humidified incubator (21% O2, 5% CO2). For macrophage differentiation, F4/80 cell surface marker (Invitrogen, 12-4801-80) was used according to manufacturer's instruction. For mitochondrial mass and membrane potential measurements, cells were incubated with MitoTracker Green FM (M7514, ThermoFisher Scientific) or Tetramethyl rhodamine Methyl Ester Perchlorate (TMRM, 11550796, Invitrogen) according to the manufacturer's instructions. Cells were then washed twice with PBS, scraped and resuspended in DMEM with FBS (1%) prior to analysis.
Instrument	BD LSRFortessa™ Cell Analyzer
Software	Data was analysed using FlowJo software version 10.10.0 (FlowJo LLC).
Cell population abundance	No sorting was done.
Gating strategy	Gating strategy is shown in Extended data figure 2f.

☒ Tick this box to confirm that a figure exemplifying the gating strategy is provided in the Supplementary Information.

This checklist template is licensed under a Creative Commons Attribution 4.0 International License, which permits use, sharing, adaptation, distribution and reproduction in any medium or format, as long as you give appropriate credit to the original author(s) and the source, provide a link to the Creative Commons license, and indicate if changes were made. The images or other third party material in this article are included in the article's Creative Commons license, unless indicated otherwise in a credit line to the material. If material is not included in the article's Creative Commons license and your intended use is not permitted by statutory regulation or exceeds the permitted use, you will need to obtain permission directly from the copyright holder. To view a copy of this license, visit http://creativecommons.org/licenses/by/4.0/
